# Invasive lionfish had no measurable effect on prey fish community structure across the Belizean Barrier Reef

**DOI:** 10.7717/peerj.3270

**Published:** 2017-05-25

**Authors:** Serena Hackerott, Abel Valdivia, Courtney E. Cox, Nyssa J. Silbiger, John F. Bruno

**Affiliations:** 1 Department of Marine Sciences, University of North Carolina at Chapel Hill, Chapel Hill, NC, United States of America; 2 STEM Department, The College of the Marshall Islands, Majuro, Marshall Islands; 3 Department of Biology, University of North Carolina at Chapel Hill, Chapell Hill, NC, United States of America; 4 Oceans Program, Center for Biological Diversity, Oakland, CA, United States of America; 5 Smithsonian Marine Conservation Program, Smithsonian Institution National Museum of Natural History, Washington, DC, United States of America; 6 Department of Ecology and Evolutionary Biology, University of California, Irvine, Irvine, CA, United States of America

**Keywords:** Belize, Community composition, Invasive species, Coral reefs, Lionfish, Belize Barrier Reef, Predators, Species diversity, Predator-prey, *Pterois volitans*

## Abstract

Invasive lionfish are assumed to significantly affect Caribbean reef fish communities. However, evidence of lionfish effects on native reef fishes is based on uncontrolled observational studies or small-scale, unrepresentative experiments, with findings ranging from no effect to large effects on prey density and richness. Moreover, whether lionfish affect populations and communities of native reef fishes at larger, management-relevant scales is unknown. The purpose of this study was to assess the effects of lionfish on coral reef prey fish communities in a natural complex reef system. We quantified lionfish and the density, richness, and composition of native prey fishes (0–10 cm total length) at sixteen reefs along ∼250 km of the Belize Barrier Reef from 2009 to 2013. Lionfish invaded our study sites during this four-year longitudinal study, thus our sampling included fish community structure before and after our sites were invaded, i.e., we employed a modified BACI design. We found no evidence that lionfish measurably affected the density, richness, or composition of prey fishes. It is possible that higher lionfish densities are necessary to detect an effect of lionfish on prey populations at this relatively large spatial scale. Alternatively, negative effects of lionfish on prey could be small, essentially undetectable, and ecologically insignificant at our study sites. Other factors that influence the dynamics of reef fish populations including reef complexity, resource availability, recruitment, predation, and fishing could swamp any effects of lionfish on prey populations.

## Introduction

Exotic predators can have striking effects on native communities (Simberloff, 1981; Bruno et al., 2005; Sax et al., 2007). For example, the introduction of brown tree snakes to the island of Guam caused the local extinction of at least a dozen bird species (Wiles et al., 2003). Likewise, the addition of the Nile perch to Lake Victoria, and of bass and trout to thousands of lakes in North America, have had similar effects on native fishes and invertebrates (Gido & Brown, 1999; Beisner, Ives & Carpenter, 2003; Eby et al., 2006). Yet these and other well-known case studies of extreme impacts are typically from isolated communities with limited connectivity, e.g., lakes and islands. The general effects of exotic predators on open ecosystems, including in marine communities, is less clear.

Indo-Pacific lionfish (*Pterois volitans* and *Pterois miles*, hereafter called “lionfish”) were introduced to south Florida in the mid-1980’s and became established and common throughout the Greater Caribbean only ten years later (Schofield, 2009). Several characteristics such as cryptic coloration (Kindinger, 2014), undetectable chemical cues (Lönnstedt & McCormick, 2013), and novel predation tactics (Albins & Lyons, 2012; Anton et al., 2016), are believed to increase their predatory efficiency in the invaded range. These traits combined with high densities and their generalist diet—consuming over a hundred Caribbean fish and invertebrate species (Morris & Akins, 2009; Green et al., 2012; Green & Côté, 2014)—suggest that lionfish are capable of causing substantial changes in native fish communities (Green et al., 2012). Based on these characteristics, Albins & Hixon (2011) described a “worst case scenario in which most reef-fish biomass was converted to lionfish biomass, leaving invaded reefs depauperate of native fishes”. Similarly, a modeling study based on Ecopath-with-Ecosim suggested that the biomass of small and medium-size carnivorous–omnivorous fishes could decline after 15 years following the lionfish invasion (Arias-González et al., 2011). However, evidence for such extreme effects is largely based on small-scale experiments using artificial reefs and artificially high lionfish densities (Albins & Hixon, 2008; Albins, 2013; Albins, 2015). In fact, a relationship between reef fish communities and natural lionfish densities has not been detected on natural reef habitats (Elise et al., 2014). Additionally, whether the effects observed on small and isolated patch reefs occur on contiguous reefs or at larger management-relevant scales is unknown.

The effects of lionfish on native prey in small-scale experiments (e.g., cages or artificial reefs of <1–4 m^2^) may not necessarily reflect outcomes in nature. Small-scale experiments have shown predatory reef fishes can have strong effects on prey population and community dynamics. For example, natural levels of post-settlement mortality of recently settled fishes are high with over 50% of individuals eaten during the first 1–2 days after settlement (Almany & Webster, 2004). Small-scale studies of prey community response to predation suggest piscivores can alter community composition, either by increasing (Webster & Almany, 2002) or decreasing (Almany & Webster, 2004) prey diversity depending on the density and identity of predators. However, due to the high mobility of predatory fishes, it is difficult to document the degree to which small-scale and short-term fish dynamics apply to larger scales (i.e., kilometers) and longer time scales more relevant to management and conservation. Therefore, large-scale monitoring of prey population and community response before and after lionfish invasion will not only provide insight into the effects of lionfish, but also a unique “natural” experiment to understand the response of native fish communities to shifts in predation pressure.

The purpose of this study was to measure the realized effects of the lionfish invasion on reef fish communities and whether reported short-term effects from controlled settings and at small spatial scales (Albins & Hixon, 2008; Green et al., 2012; Albins, 2013) are evident at larger scale reef habitats. We quantified the relationship between lionfish density and the density, richness, and community composition of small native prey fishes at 16 sites along the Belizean Barrier Reef (BBR). Lionfish were first documented in Belize in 2008 (Schofield, 2009; Schofield, 2010). Our study began in 2009, with no lionfish present on our study sites, and continued until 2013. Due to the timing of our study, we were able to track changes in the community of small reef fish from before lionfish invaded our sites, during the onset of the invasion of our sites in 2010, and for two years after the invasion in 2012 and 2013. Based on previous studies we hypothesized that: (1) the density and species richness of small potential prey fish would be negatively related to lionfish density due to predation; and (2) that there would be a shift in small reef fish community composition because lionfish differentially reduce prey density (Green et al., 2012) by selectively feeding on species with certain morphological and behavioral traits (Green & Côté, 2014).

## Methods

### Study sites and reef fish surveys

Our longitudinal study began during the onset of the lionfish invasion in Belize in 2009 (Schofield, 2009) and continued until 2013. We used a modified-BACI design (Before-After-Control-Impact) as we tracked changes in native fishes among sites as lionfish density varied over time. The “Before-After” aspect of the project was accomplished by surveying sites before and after they were invaded by lionfish. For this study, we considered a site to have been “invaded” when the measured density of lionfish was >0. The modified aspect was that the “Control-Impact” categorization was continuous (i.e., based on lionfish density) rather than categorical as in a traditional BACI design (i.e., we did not base the analysis on lionfish presence/absence).

**Figure 1:**
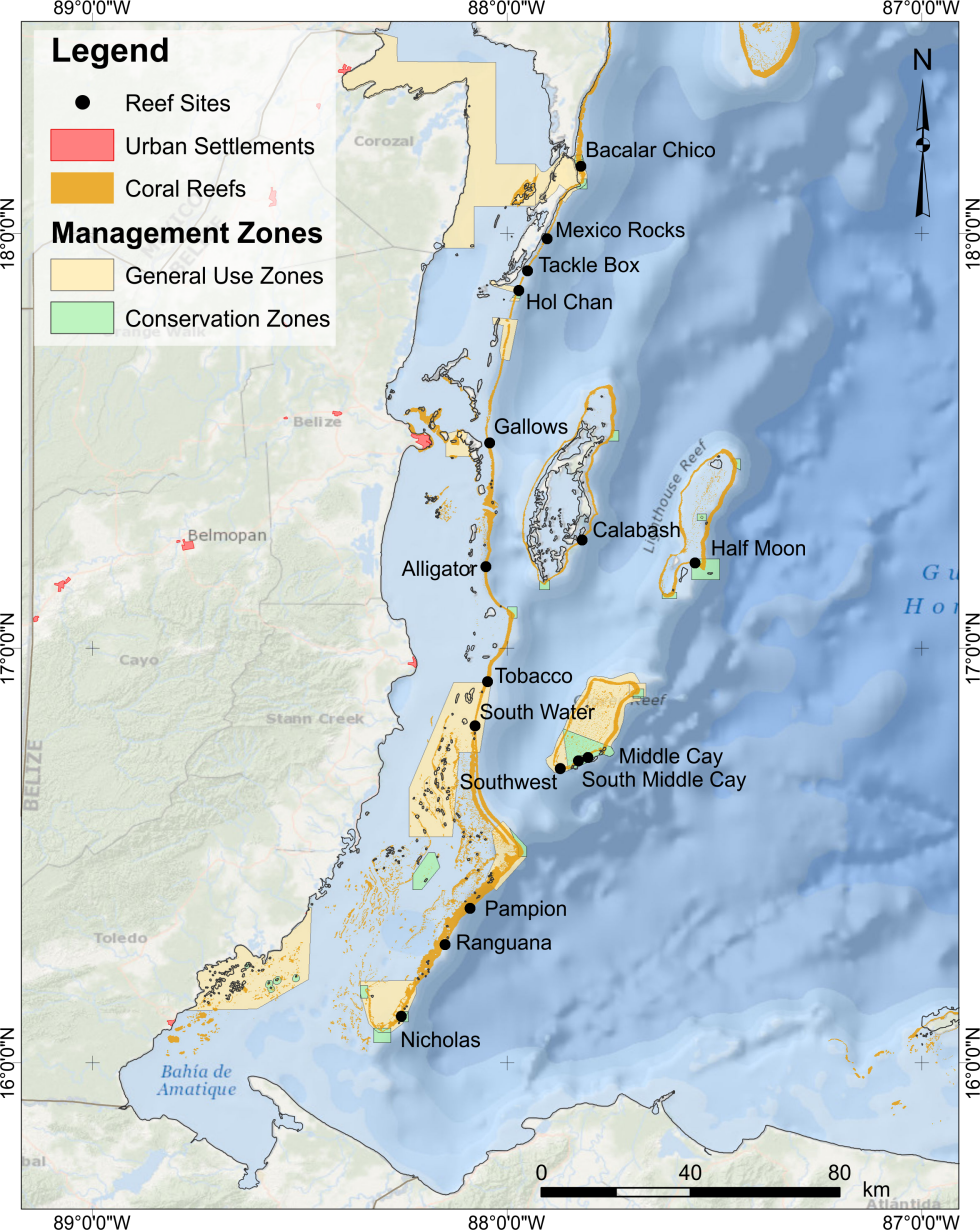
Location of survey sites. For sites abbreviations, coordinates, and other site info, refer to Table S1.

We monitored 16 fore-reef sites at 12–15 m depth, spanning ∼250 km, across gradients of natural environmental conditions (Fig. 1, Table S1). To minimize habitat variability of study sites, we only surveyed spur-and-groove habitats formerly dominated by the reef building corals *Orbicella* spp. At each site, we performed underwater visual censuses of reef fish using six to eight belt transects placed along the spur-and-groove formations. We counted and identified reef fish to species and estimated total length (TL) in 5–10 cm intervals (McClanahan et al., 2007; McClanahan & Humphries, 2012; McClanahan, 2014). Divers counted fish less than 5 cm in TL in 15 × 1 m belt transects and fish between 6 and 40 cm TL in 30 × 2 m belt transects. Fish >40 cm TL as well as lionfish were counted in 50 × 10 m belt transects. Lionfish and large fish counts included a thorough search for cryptic individuals within the surveyed area. At each site, the longer and wider transect (50 × 10 m) encompassed the smaller transects (30 × 2 m). One diver surveyed fish <40 cm TL while laying the transect. After completing the 30 × 2 m survey, this diver continued to lay the same transect line for a total length of 50 m. A separate diver followed behind to survey lionfish and large predators, swimming in a zig-zag pattern, surveying the benthos as well as the water column for both benthic and pelagic species >40 cm TL as well as lionfish. Fish surveyors were consistent across years to avoid surveyor-bias. Sites were surveyed once per year in 2009, 2010, 2012, and 2013 from mid-May to early June. Due to logistical constraints, surveys were not conducted in 2011. Field work was approved by the Belize Fisheries Department (permit numbers 000028-11 and 000018-09).

### Reef fish community structure

We quantified changes in reef fish density, richness, and community composition for all small individuals that are potential prey for lionfish. We defined potential prey based on the sizes of lionfish on our sites. Of the 143 lionfish recorded on our sites throughout the study, ∼80% were >20 cm in TL (Fig. S1). As lionfish likely consume prey less than half their own body length (Albins & Hixon, 2008; Morris & Akins, 2009), we determined that potential prey were likely within the 6–10 cm TL size class, and therefore focused our analysis on this range. We further defined potential prey fish as species documented as lionfish prey on comparable Caribbean habitats (Albins & Hixon, 2008; Morris & Akins, 2009; Layman & Allgeier, 2011; Green et al., 2012; Valdez-Moreno et al., 2012; Albins, 2013; Côté et al., 2013; Green & Côté, 2014; Dahl & Patterson, 2014; Rocha et al., 2015) (Table S2). Although densities of fish <5 cm in TL are potentially too variable at the scale of our study for meaningful conclusions, we have included models of the abundance of this size class in the supplement (Text S1).

### Reef complexity

We included reef complexity as a covariate in our analysis because highly complex reefs have been shown to support higher reef fish abundances or diversity compared with less complex reefs (Friedlander et al., 2003). Habitat complexity can also potentially influence predator–prey interactions (and per capita predation intensity) (Crowder & Cooper, 1982; Persson & Eklov, 1995; Beukers & Jones, 1998). However, based on previous studies, we did not expect reef complexity to influence lionfish abundance (Anton, Simpson & Vu, 2014; Valdivia et al., 2014). To estimate reef structural complexity, we used a semi-quantitative index from 0 to 5, where “0” was a reef with no vertical relief and “5” was an exceptionally complex reef with overhangs, crevices and deep cuts (Polunin & Roberts, 1993; Valdivia et al., 2014). Reef complexity was visually estimated along each transect and then averaged to obtain a single value for each site (Table S1).

### Analysis of the effects of the lionfish invasion

Small reef fish density (individuals/m^2^) was calculated for each prey species, and summed to obtain the total density of small prey fish. Species richness for small prey fish was calculated as the total number of species present on each transect, excluding lionfish, at each site for each year.

We used generalized linear mixed effect models (GLMM) to evaluate the response of two reef fish community metrics (e.g., density and richness) of all potential prey species 6–10 cm TL, as well as the density of small prey fish within each of the most abundant families, to lionfish density (individuals/ha), with year and site-specific reef complexity as co-variates. Additional models were ran with the density of 0–5 cm TL and 0–10 cm TL prey fish as response variables (Text S1). Reef fish community responses (density and richness) were averaged over transect for each site and each year and all predictor variables were standardized (centered and divided by standard deviation) in all models. Transect-level data were highly zero-inflated and did not meet assumptions of a GLMM; however, we have included transect-level models in the supplement (Text S2). We used a Gamma distribution with a log link function in the prey fish density model and a Gaussian distribution in the prey fish richness model, both using the *lme4 (v1.1-5)* package in R (Bates et al., 2014, p. 4). Lionfish density, year, and reef complexity were included as fixed effects in each model. Site was included as a random effect in all models to account for variability across sites.

In addition to using lionfish as a continuous variable in our models (which assesses the relationship between lionfish density and prey density), we also ran similar models for prey fish density with lionfish as a discrete variable, splitting lionfish density into “low” or “high” levels. We identified sites in each year with “high” lionfish densities as those with average lionfish densities greater than the approximate maximum native density of 25 individuals/ha (Kulbicki et al., 2011; Darling et al., 2011; Cure, McIlwain & Hixon, 2014; McTee & Grubich, 2014). We also repeated this analysis using threshold values of 20 individuals/ha, 15 individuals/ha, and 10 individuals/ha to test the effect of lionfish on prey fish density at a range of thresholds.

To check for homogeneity of variance of the model residuals, we visually inspected plots of the residuals against the fitted values and each of the predictors. To test for collinearity in our explanatory variables, we used a Spearman’s correlation matrix and calculated the variance inflation factor (VIF) for each predictor; a threshold of VIF < 2 was used to determine if variables were significantly correlated (Graham, 2003). Quantile–quantile plots *(car package v2.0-19)* were used to check for normality of the model residuals (Fox & Weisberg, 2011). Spline-correlograms *(ncf package v1.1-5)* using the spatial coordinates of each site with 1,000 resamples revealed no evidence of spatial autocorrelation of the residuals of each final model (Bjornstad, 2013).

We quantified small prey fish community composition in terms of Bray–Curtis dissimilarity. Changes in prey fish community composition in response to year and lionfish density were assessed using permutational multivariate analysis of variance (PERMANOVA) of transect-level data with 10,000 permutations, stratified by site (*vegan package v2.0-10)* (Oksanen et al., 2013). Prey fish community composition was visualized with a nonmetric multidimensional scaling (NMDS) analysis, using both species density and species presence/absence data, at the site level. Both density and presence/absence were analyzed to determine if changes in community composition were driven by changes in species identity or abundance. For both analyses, we visualized changes in community composition over time by drawing ellipses of the standard deviation around the centroids for each year using the ordiellipse function (*vegan package v2.0-10)* (Oksanen et al., 2013).

## Results

No lionfish were recorded on any of our study sites in 2009. Lionfish were present on two sites (Calabash and South Middle Caye, Fig. S2) at relatively low densities (mean ± SE, 6.7 ± 6.7 individuals/ha on both sites) in 2010. By 2012, lionfish were observed on 14 of 16 surveyed sites and on all sites by 2013. Across all sites, the average lionfish density (mean ± SE) increased from 0.9 ± 0.7 individuals/ha in 2010 to 16.3 ± 5 individuals/ha in 2012, and dropped to 11.1 ± 4.2 individuals/ha in 2013. The highest lionfish density found during our study was 70 ± 29.2 individuals/ha at Pampion in 2012. Lionfish density varied among sites over time (Fig. S2).

**Figure 2:**
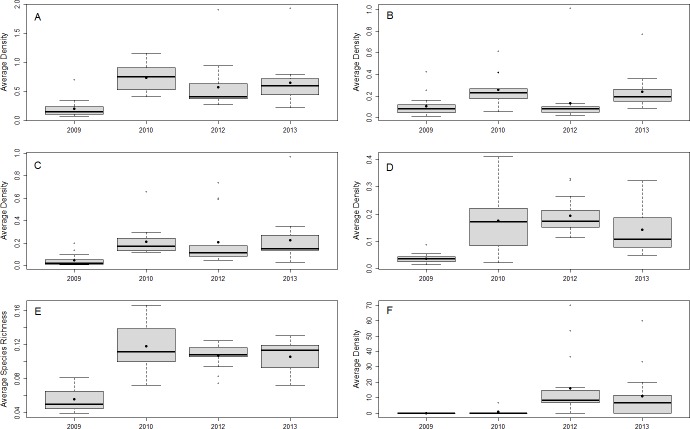
Small fish community responses and lionfish abundance over time. Boxplots of site-averaged total abundance (individuals/m^2^) (A), family-specific abundance (individuals/m^2^) (Pomacentridae, Labridae, and Scaridae B–D, respectively), and species richness (species/m^2^) (E) of prey small fish, as well as lionfish abundance (individuals/ha) (F) for each survey year. Boxes indicate the first and third quartiles with the median shown by the thicker transecting line. Mean values are indicated by the filled black dots.

From 2009 to 2013, 46 species of fish in the 0–5 cm size class and 77 species of fish in the 6–10 cm TL size class were observed, 33 of which have been previously documented as prey species for lionfish (Table S2). The most abundant documented lionfish prey in the 6–10 cm size class were damselfish (Pomacentridae), wrasses (Labridae), and parrotfish (Scaridae), accounting for ∼34%, ∼33%, and ∼26% of the total density of small prey fish in this size class, respectively. The density and species richness of all potential prey fish, as well as the density of the most abundant families, fluctuated over time, but were generally higher after 2009 (Fig. 2).

**Figure 3:**
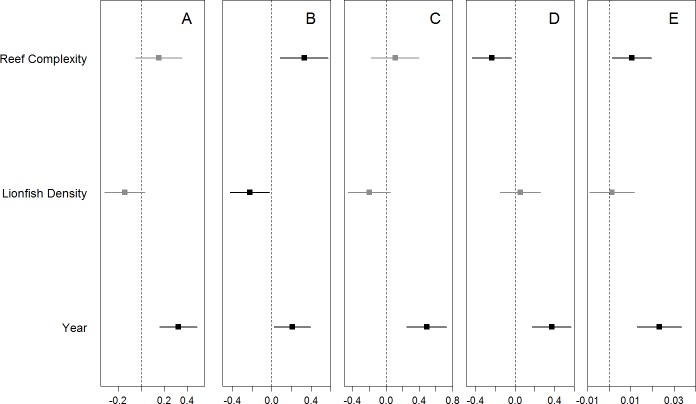
Coefficient estimates (mean ± 95% confidence interval) for each model. The site-averaged abundance of all potential prey fish (A), the abundance of the most common families (Pomacentridae, Labridae, and Scaridae B–D, respectively), as well as species richness (E) of potential prey fish were each modeled with the predictors of interest (lionfish abundance and years since the lionfish invasion) as well as site-specific reef complexity. Significant coefficient estimates are shown in black while non-significant coefficients are shown in gray (details in Table S3).

We found no significant relationship between lionfish density and total density and species richness of potential prey fishes, or the density of small wrasses and parrotfish (Fig. 3, Table S3). The one exception was a marginal negative relationship between the densities of damselfish and lionfish (Fig. 3, Table S3). Total and family-specific density and species richness of all potential prey fish increased over time (Fig. 3, Table S3). The effect of year was ∼2–5 times greater than the effect of lionfish density on the total density and species richness of prey fishes and the density of small wrasses and parrotfish (Table S3). The density of small damselfish and parrotfish, as well as the total species richness of potential prey fish, were related to reef complexity, where species richness and damselfish density increased, while parrotfish density decreased with increasing reef complexity (Fig. 3, Table S3). Similarly, none of the prey fish responses were related to lionfish densities when transect level data was used, and year had a positive effect of ∼2–11 times greater than that of lionfish density (Text S2, Table S3). In addition, the density of small prey fish was not significantly different between “high” and “low” lionfish densities, regardless of the categorical threshold density used (25–10 individuals/ha) (Fig. S3). Results were similar for models of prey fish <5 cm TL when modeled separately, or combined with prey fish 6–10 cm with year having a positive effect ∼2–3 times greater than lionfish density while lionfish density was not related to prey fish density (Text S1, Fig. S4, Table S4).

**Figure 4:**
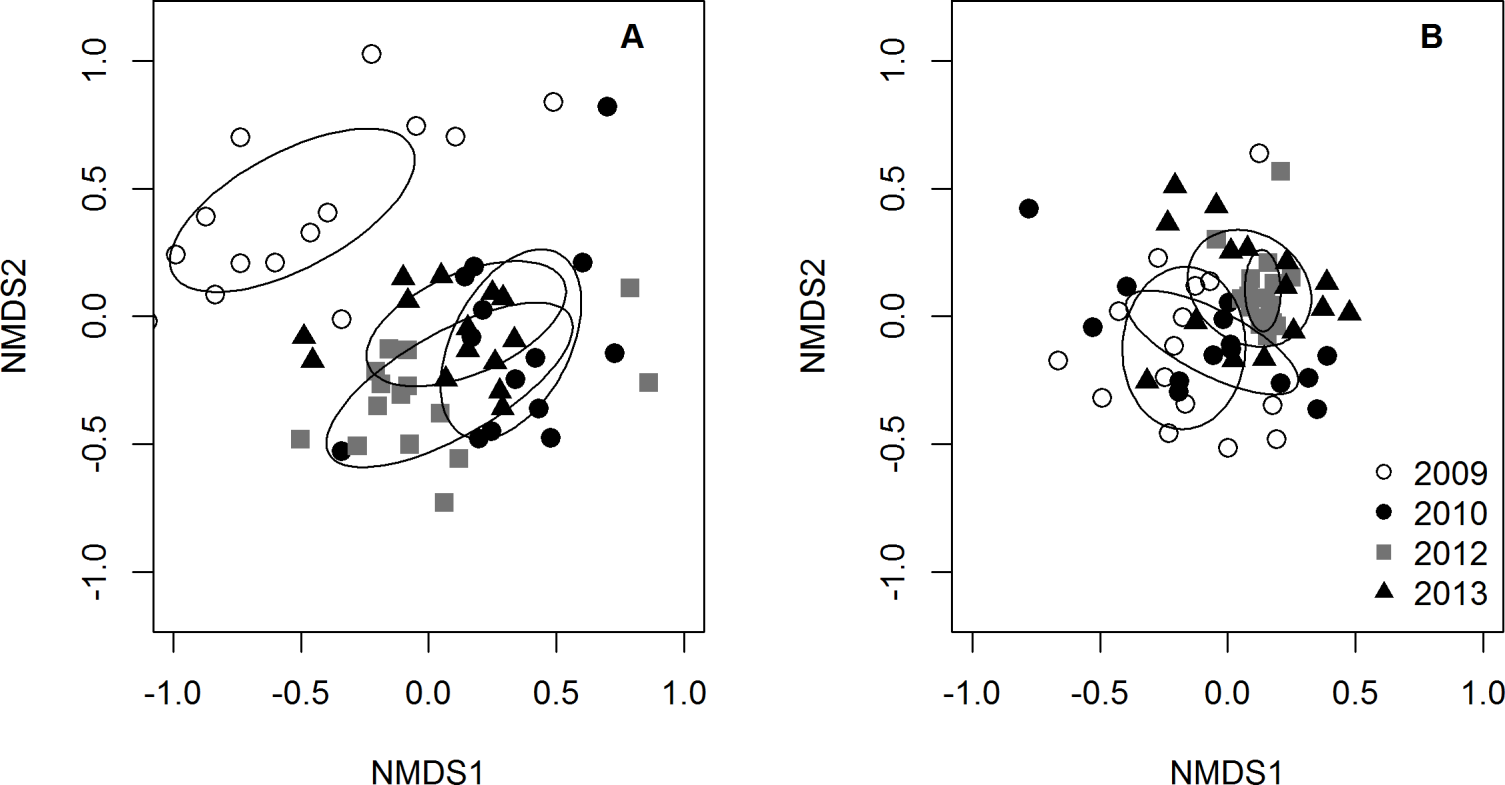
NMDS plots of small prey fish community composition. Community composition was visualized using (A) prey species abundance (density) and (B) prey species presence/absence. Each point represents the average community composition across all transects from each site for each year surveyed. Ellipses are standard deviations of the mean for each year. When using density data, 2009 is distinct from all other years (A) whereas there are no differences between years when using presence/absence data (B).

The PERMANOVA analyses indicated that the community composition of potential prey species changed over time (*p*-value = 1.0e^−6^), but was not related to lionfish density (*p*-value = 0.54). The NMDS plot of prey species abundance showed that prey fish communities in 2009 were substantially different from prey communities in 2010, 2012, and 2013, which all clustered together (Fig. 4A). However, this separation was not observed on the NMDS plot of prey species using presence/absence data (Fig. 4B), indicating that there was a change prey abundance over time rather than a change in species identity.

## Discussion

Lionfish density varied greatly across years and sites during our four year, sixteen site study (Fig. S2). We used this variation as a “natural experiment” (i.e., as lionfish density treatments) via a modified BACI design to quantify the effects of invasive lionfish on the community structure of 33 native prey species within the context of natural environmental variability. Despite the broad assumption that lionfish are one the greatest threats to Caribbean reefs, we found no evidence of a general, negative effect on native prey fishes. In fact, total small prey density and species richness *increased* after 2009 (Fig. 2). We also found that species composition shifted over time due to a temporal change in abundance, rather than a change in presence/absence patterns (Figs. 4A–4B). As Belizean reefs are classified as a larval sink (Roberts, 1997), this shift in prey fish abundance could have been due to a larval settlement pulse that coincided with the arrival of lionfish to the region. Alternatively, inter-annual variability in abiotic factors could have increased post-settlement survival (i.e., recruitment) in 2010.

Our finding that lionfish had no apparent effect on native prey communities four years post-invasion is not concordant with most past work. For example, several small-scale manipulations on artificial reefs or isolated patch reefs have documented effects on prey density (Albins & Hixon, 2008; Albins, 2013; Albins, 2015), as have at least two uncontrolled (i.e., the design did not include sites without lionfish for comparison) single site observational studies (Lesser & Slattery, 2011; Green et al., 2012). In contrast, the only previously published, controlled study quantifying community-level responses to naturally occurring lionfish densities on coral reef habitats found the density and richness of native fish assemblages did not change along ∼1.5 km of reef within the marine protected area of Archipelago Los Roques National Park in Venezuela (Elise et al., 2014).

There are at least four explanations for why the results of lionfish impact studies have been so variable. First, lionfish density–which varies substantially among studies–could strongly influence the effects on native prey and thus study outcomes. Many of the small-scale experimental tests of whether lionfish affects prey populations have used unnaturally high lionfish densities as treatments. For example, Albins (2013) added one lionfish to each replicate 4 m^2^ artificial reef, which is equivalent to 2,500 individuals/ha—nearly an order of magnitude greater than the Caribbean record (393.3 ± 144.4 individuals/ha, mean ± SD, Green & Côté, 2014) and ∼150 times higher than average post-invasion density (16.3 ± 5 individuals/ha) across the BBR (this study). Likewise, using densities of 10,000 and 3,333 lionfish/ha, Albins & Hixon (2008) found a large decline (79%) in the recruitment of native fishes compared to artificial reefs without lionfish. Besides being an extreme and unrealistic treatment level, such lionfish densities would not be stable because prey would quickly be depleted. Less extreme lionfish densities, experimentally maintained at the maximum recorded density (∼300/ha), resulted in more modest declines in abundance and richness of small prey (<10 cm TL) and no effect on 10–20 cm (TL) prey (Albins, 2015). And in Los Roques National Park, Venezuela, natural lionfish densities of 121 ± 164 individuals/ha had no effect on native prey communities (Elise et al., 2014). Likewise, we found no lionfish effects in our regional scale study, in which average post-invasion density was even lower.

The effects of lionfish likely become apparent at different densities (i.e., thresholds), depending on the prey fish community or reef habitat. On patch reefs, the effect of lionfish on the biomass of small prey individuals can be limited if lionfish densities are reduced below a reef-specific threshold (Green et al., 2014). The lionfish densities across our sites post-invasion (2012 and 2013) were lower than the threshold of lionfish density (mean ± SE; ∼138.9 ± 14.7 individuals/ha) predicted to prevent declines in prey fish on patch reefs in Eleuthera, Bahamas (Green et al., 2014). While threshold densities surely vary across habitats, site-specific “thresholds” for our larger fore-reef sites are likely higher than those estimated for smaller patch reefs. This is because reef fish communities are naturally more variable (Mellin et al., 2010) and may be more strongly structured by recruitment on isolated patch reefs than on contiguous reef systems (Ault & Johnson, 1998). Therefore, it is likely that lionfish densities on our sites were lower than the site-specific “threshold” levels, which could explain why the predicted effects of lionfish were not apparent in our study. Clearly, had densities been far greater (e.g., >300/ha) across the fore-reefs of the BBR we may have seen larger effects on native prey.

**Figure 5:**
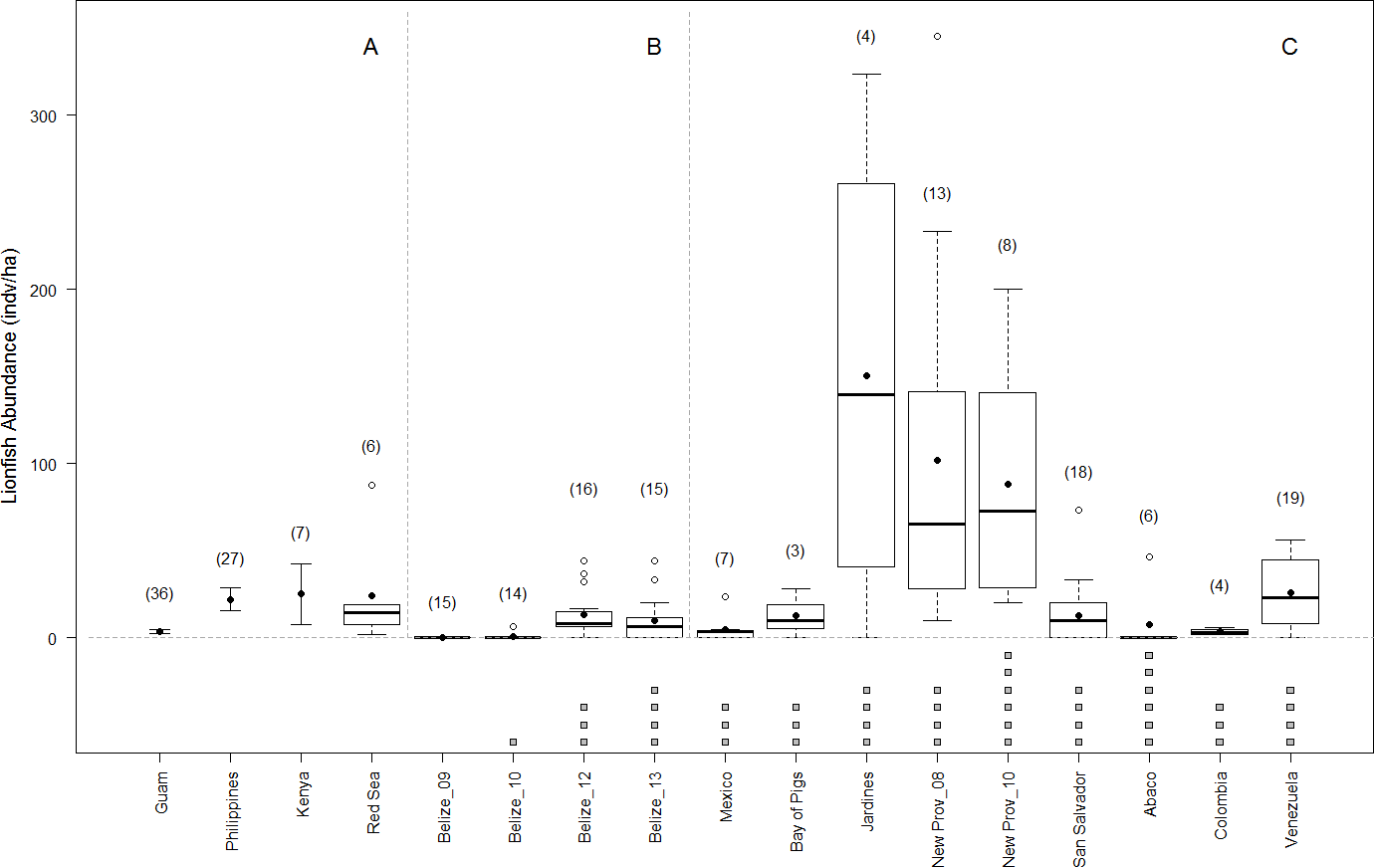
Lionfish abundance on reefs in native and invaded range. Lionfish abundance values published for similar reef habitats and similar depth ranges across the native (A) and invaded range (this study, and other studies B–C, respectively) showing boxplots of site-level data when available. Averages are shown in solid circles. When site-level data was not available, averages are transected by error bars showing published standard deviation. Number of sites surveyed within each region are shown in parentheses. Below the axis, grey boxes indicate number of years since the invasion at the time of the survey of invaded regions. Sources for lionfish density values include (Kulbicki et al., 2011; Agudo & Salas, 2014; Cure, McIlwain & Hixon, 2014; Valdivia et al., 2014; Bayraktarov et al., 2014; McTee & Grubich, 2014; Anton, Simpson & Vu, 2014).

Shortly following the arrival of lionfish in Belize, various management actions were initiated including awareness programs, lionfish fishing tournaments, and market-based approaches (Searle, Chacon & Bach, 2012; Chapman et al., 2016). It is possible that these lionfish management strategies have helped control lionfish densities in Belize, at least at sites frequently visited by tourists and dive guides (Searle, Chacon & Bach, 2012). However our observed lionfish densities along the BBR are not atypical. In fact, lionfish densities in Belize are comparable to those reported on similar reef habitats in Mexico, Cuba (Bay of Pigs), The Bahamas (San Salvador and Abaco), Colombia, and Venezuela (Agudo & Salas, 2014; Valdivia et al., 2014; Bayraktarov et al., 2014; Anton, Simpson & Vu, 2014), and may be more representative of the Caribbean region than the often-cited densities of New Providence, Bahamas (Green & Côté, 2009) (Fig. 5). Future experiments should focus on realistic density values to better determine the density-dependence of lionfish predation effects (Green et al., 2014) and the threshold below which local management actions (e.g., culling) are likely unnecessary.

Second, lionfish effects could be habitat dependent. Lionfish have invaded a wide range of habitat-types and depth ranges including estuaries, seagrass beds, mangroves, mesophotic reefs, and shallow patch reefs (Barbour et al., 2009; Claydon, Calosso & Traiger, 2011; Jud et al., 2011; Nuttall et al., 2014; Layman, Jud & Nichols, 2014), yet their impacts in these and other invaded habitats is largely unknown (although see an example on one mesophotic reef in Lesser & Slattery, 2011). It is possible that the relative effect of lionfish varies among habitats and could be greater where lionfish densities are especially high, e.g., on deep reefs (Claydon, Calosso & Traiger, 2011; Lesser & Slattery, 2011; Nuttall et al., 2014; Pinheiro et al., 2016), or where juvenile fish recruit, i.e., mangroves and seagrass beds. Even independent of density, the effects of lionfish could vary among habitat types due to differences in benthic complexity and structure and thus the availability of refugia for prey. Unlike previous shallow-water experiments (mainly performed on sand flats or patch reefs), our study was on deeper, far more physically complex and contiguous fore-reef environments. Effects are also likely dependent on prey productivity, recruitment or immigration from adjacent habitat patches. Most studies reporting effects on native communities have been performed in relatively homogeneous and isolated shallow, patch reef habitats—likely increasing predation and reducing immigration rates relative to a typical fore-reef environment. This could exaggerate predation effects and could explain the contrasting outcomes of different approaches, e.g., effects of lionfish on prey populations could be offset in more connected habitats by recruitment or immigration from surrounding areas (Ault & Johnson, 1998).

The third explanation is that other biotic and abiotic factors that influence prey population dynamics could have stronger effects than lionfish predation in a natural heterogeneous setting. Prey populations are naturally regulated by bottom-up forces such as resource availability, top-down forces such as predation, inter- and intra-specific competition, and stochastic processes such as variability in recruitment or environmental disturbances (Sale, 1977; Shulman, 1984; Jones, 1986; Hunter & Price, 1992; Power, 1992). For example, our results indicate habitat complexity had up to five times greater effect on the density and richness of some families of small reef fishes than lionfish densities. Additionally, we found that the species richness and total prey density, as well as the density of small damselfish, wrasses, and parrotfish increased over time, suggesting that reef fish communities were more strongly structured by inter-annual variability (e.g., annual changes in recruitment or environmental conditions) than by lionfish predation. Such spatiotemporal heterogeneity is generally eliminated in a small-scale, short-term experiment, e.g., where all replicate habitat patches are identical, all others factors that could influence prey population dynamics are held constant, etc. Although these experiments allow us to evaluate the potential role of a given factor, inferences that can be drawn from the results are limited. Such experiments test whether lionfish *could* affect prey communities, not whether they actually do within natural contexts. For example, during experiments that demonstrated a significant effect on reef fish recruits, lionfish comprised ∼50–100% of the total predator biomass (Albins & Hixon, 2008; Albins, 2013). In contrast, in our study lionfish were only ∼10% of the total potential predator biomass post-invasion (in 2012 and 2013) (Fig. S5, Table S5, Text S3).

The relative importance of lionfish predation compared to natural abiotic and biotic factors in structuring reef fish communities may vary between contexts. For example, lionfish had no detectable effect on reef fish communities on healthy reef fish communities in the Archipelago Los Roques National Park in Venezuela (Elise et al., 2014), suggesting that lionfish are less likely to have apparent impacts in more intact reef ecosystems. The Belizean Barrier Reef is protected by one of the most extensive networks of marine protected areas in the Caribbean (Healthy Reefs Initiative, 2014). These protected reefs may therefore have healthier reef fish assemblages (although see Cox et al., 2017) and be less likely to be measurably affected by invasive lionfish compared to more degraded reef systems.

The fourth, and more overarching, potential explanation is that the effects of lionfish may vary with time since invasion in combination with previously discussed factors. For example, the threshold density at which lionfish measurably effect prey fish is influenced by both lionfish density and prey productivity (Green et al., 2014). Therefore, the impacts of lionfish may become apparent if prey productivity decreases or lionfish density increases over time on our study sites. Additionally, a longer time period may be required for impacts of lionfish to become apparent in certain contexts such as complex habitats with ample prey refugia, interconnected reef systems, larval sink ecosystems, or ecosystems with healthy, intact native communities.

In summary, our results suggest that fish communities appear unaffected by lionfish across the sites surveyed along the BBR. In contrast to the outcome of small-scale experiments, lager-scale field studies based on BACI designs have failed to detect negative effects of lionfish predation on native reef fish communities. This could be due to differences in scale, habitat, location, or lionfish density. Future studies of lionfish impacts should focus on quantifying the context dependency of impacts across a range of habitats and lionfish densities, and ideally relative to other threats including fishing, pollution, and climate change.

##  Supplemental Information

10.7717/peerj.3270/supp-1 Supplemental Information 1 Appendix: Supplemental Text, Tables, and Figures

## Additional Information and Declarations

### Field Study Permissions

The following information was supplied relating to field study approvals (i.e., approving body and any reference numbers): Field work was approved by the Belize Fisheries Department (permit numbers 000028-11 and 000018-09).

### Data Availability

The following information was supplied regarding data availability: GitHub: https://github.com/johnfbruno/Rcode-and-data-for-Hackerott-et-al.
