# Supplementary material for: Invasive lionfish had no measurable effect on prey fish community structure across the Belizean Barrier Reef
Source: PeerJ. 2017 May 25;5:e3270. doi: 10.7717/peerj.3270 (PMC5446774; doi:10.7717/peerj.3270)
Supplement: Supplemental Information 1 [file peerj-05-3270-s001.docx]

**Appendix**

*Supplemental Text*

**Text S1. Analysis of Prey Fish 0-5cm TL**

The site-level abundance of small prey fish less than 5cm in total length, as well as the abundance of small prey fish 0-10cm TL were modeled using generalized linear mixed effect models (GLMM). A Gamma distribution with a log link function was used with the *lme4 (v1.1-5)* package in R (Bates et al., 2014, p. 4) for both models. Lionfish density, year, and reef complexity were standardized (centered and divided by standard deviation) and included as fixed effects in each model. Site was included as a random effect in all models to account for variability across sites. Prey species less that 5cm in total length were identified as species documented as lionfish prey on comparable Caribbean habitats (Table S2), with the addition of “Gobiidae” for individuals in the Gobiidae family that were not identified to the genus or species level.

**Text S2. Transect-Level Analysis Information Differing From Site-Level Analysis**

Reef fish community responses (abundance and richness) of the total prey fish and the most dominant families were modeled using the *glmmADMB (v0.8.0)* package with a negative binomial distribution and log link. Extreme outliers greater than the 99^th^ percentile of lionfish abundance and outliers greater than the 97-99^th^ percentile of the total and family-specific abundance were removed in each model to aid with model fit. Counts were used as the response variable for the models of both abundance and richness. Counts of abundance were offset by log(survey area) so that the response variable was density of fish (individuals/m^2^). The residuals of the models of reef fish abundance were non-normal, however these were the best fit models for our transect-level data.

**Text S3.** **Lionfish and Competitor Biomass Calculations**

Lionfish competitor species (Table S4) were identified as predatory species that were ecologically similar to lionfish based on diet from Green et al. (2012) and FishBase (R Froese and Pauly 2011). Biomass for lionfish and each competitor species was calculated through the allometric length-weight conversion formula, W = aTLb, where W is the weight of each individual fish in grams, TL is the total length recorded for each fish in cm, and the parameters a and b are species-specific (R Froese and Pauly 2011). We used the mid-point of the 10 cm TL-estimate intervals to calculate biomass. The variables a and b were obtained from FishBase (R Froese and Pauly 2011), selecting the values from areas that were geographically closest to Belize. When these variables were not available, we used the values of congeneric species of similar size and morphology.

*Supplemental Tables*

**Table S1. Site Information and Coordinates.**

| **Site Name** | **Site Code** | **Latitude (N)** | **Longitude (W)** | **Complexity** |
| --- | --- | --- | --- | --- |
| Alligator | AL | 17.1966 | -88.0512 | 2 |
| Bacalar Chico | BC | 18.16282 | -87.8222 | 2.92 |
| Calabash | CA | 17.26147 | -87.8197 | 2.42 |
| Gallows | GA | 17.49592 | -88.0426 | 3.5 |
| Hol Chan | HC | 17.86343 | -87.9724 | 2 |
| Half Moon | HM | 17.2056 | -87.5468 | 5 |
| Middle Cay | MC | 16.73703 | -87.8054 | 1.67 |
| Mexico Rocks | MR | 17.98782 | -87.9038 | 2.58 |
| Nicholas | NI | 16.1123 | -88.2559 | 2.33 |
| Pampion | PO | 16.3731 | -88.0891 | 2.92 |
| Ranguana | RA | 16.28501 | -88.1503 | 2.83 |
| South Middle Cay | SM | 16.72875 | -87.8287 | 2 |
| Southwest | ST | 16.11247 | -88.2711 | 2.33 |
| South Water | SW | 16.81346 | -88.0776 | 2.08 |
| Tackle Box | TB | 17.91056 | -87.9508 | 2.75 |
| Tobacco | TO | 16.91911 | -88.0476 | 2.08 |

**Table S2. Potential Prey Families and Species.** Fish species within the 6-10 cm size class documented as lionfish prey with source(s) of documentation.

| **Family** | **Species** | **Prey Species Documentation** |
| --- | --- | --- |
| Acanthuridae | *Acanthurus bahianus* | 3 |
|  | *Acanthurus chirurgus* | 6 |
|  | *Acanthurus coeruleus* | 5 |
| Cirrhitidae | *Amblycirrhitus pinos* | 3 |
| Grammatidae | *Gramma loreto* | 1,2,3,4,7 |
| Haemulidae | *Haemulon aurolineatum* | 7, 10 |
|  | *Haemulon flavolineatum* | 1,7 |
| Holocentridae | *Holocentrus rufus* | 2,7 |
| Labridae | *Bodianus rufus* | 3,4,6,7,8 |
|  | *Clepticus parrae* | 2,3,4,7 |
|  | *Halichoeres bivittatus* | 2,3,4,7,8,9,10 |
|  | *Halichoeres garnoti* | 1,2,3,4,5,7,8 |
|  | *Halichoeres maculipinna* | 2,3,4,5,6 |
|  | *Thalassoma bifasciatum* | 1,2,3,4,6,7,8 |
| Lutjanidae | *Ocyurus chrysurus* | 3,5 |
| Mullidae | *Pseudupeneus maculatus* | 3,4,7 |
| Pomacentridae | *Abudefduf saxatilis* | 1,8 |
|  | *Chromis cyanea* | 2,3,4,7 |
|  | *Chromis enchrysura* | 10 |
|  | *Chromis insolata* | 3 |
|  | *Chromis multilineata* | 2,3,4,7 |
|  | *Stegastes leucostictus* | 3,6,8 |
|  | *Stegastes partitus* | 1,2,3,4,5,6,7 |
|  | *Stegastes variabilis* | 2,3,4,5,7, 9 |
| Scaridae | *Scarus iserti* | 1,3,7,9 |
|  | *Scarus taeniopterus* | 1 |
|  | *Sparisoma atomarium* | 5,6,9 |
|  | *Sparisoma aurofrenatum* | 1,2,4,5,6,7,9 |
|  | *Sparisoma viride* | 1,3,5,6,9 |
| Serranidae | *Cephalopholis cruentata* | 1,2,4,7 |
|  | *Hypoplectrus puella* | 9 |
|  | *Serranus tigrinus* | 2,3,4,5,6,7 |
| Tetraodontidae | *Canthigaster rostrata* | 3,5,6,7 |

*Note:* Sources are 1. Valdez-Moreno et al (2012), 2. Cote et al (2013), 3. Morris and Akins (2009), 4. Green et al (2012), 5. Albins and Hixon (2008), 6. Albins (2013), 7. Green and Cote (2014), 8. Layman and Allgeier (2011), 9. Rocha et al. (2015), 10. Dahl and Patterson (2015)

**Table S3. Coefficient Estimates and p-values from GLMMs.** Coefficient estimates and p-values for models of potential prey fish abundance in the 6-10cm size class, the abundance of the most common families, and species richness using both site level and transect level data. Significant coefficient estimates are shown in bold at alpha 0.05.

| **Response** | **Transect vs Site Level** | **Year** | | **Lionfish Abundance** | | **Reef Complexity** | |
| --- | --- | --- | --- | --- | --- | --- | --- |
|  |  | *Estimate* | *p-value* | *Estimate* | *p-value* | *Estimate* | *p-value* |
| Total Abundance | Site | **0.32** | **9.74 e-5** | -0.14 | 0.11 | 0.15 | 0.14 |
|  | Transect | **0.40** | **2.3e-15** | -0.07 | 0.13 | 0.09 | 0.31 |
| Pomacentridae Abundance | Site | **0.21** | **0.03** | **-0.22** | **0.03** | **0.33** | **0.01** |
|  | Transect | **0.18** | **5.5e-3** | -0.09 | 0.15 | **0.21** | **0.03** |
| Labridae Abundance | Site | **0.49** | **<2e-16** | -0.20 | 0.13 | 0.11 | 0.45 |
|  | Transect | **0.59** | **1.7e-14** | -0.13 | 0.07 | 0.04 | 0.62 |
| Scaridae Abundance | Site | **0.37** | **2.57e-4** | -0.05 | 0.60 | **-0.24** | **0.02** |
|  | Transect | **0.53** | **3.4e-13** | -0.10 | 0.129 | **-0.17** | **0.01** |
| Species Richness | Site | **0.02** | **4.76e-5** | 0.00 | 0.80 | **0.01** | **0.03** |
|  | Transect | **0.22** | **<2e-16** | -0.02 | 0.45 | 0.04 | 0.22 |

**Table S4. Coefficient Estimates and p-values from GLMMs Including Prey Fish <5cm TL.** Coefficient estimates and p-values for models of potential prey fish abundance split by size class using site level data. Significant coefficient estimates are shown in bold at alpha 0.05.

| **Response** | **Size Class** | **Year** | | **Lionfish Abundance** | | **Reef Complexity** | |
| --- | --- | --- | --- | --- | --- | --- | --- |
|  |  | *Estimate* | *p-value* | *Estimate* | *p-value* | *Estimate* | *p-value* |
| Abundance of Small Prey Fish | 0-5cm TL | **0.40** | **2.17e-6** | -0.09 | 0.27 | 0.23 | 0.07 |
|  | 6-10cm TL | **0.32** | **9.74 e-5** | -0.14 | 0.11 | 0.15 | 0.14 |
|  | 0-10cm TL | **0.38** | **3.11e-8** | -0.11 | 0.17 | 0.21 | 0.07 |

**Table S5. Competitor Species.** Fish species present on our surveys that are ecologically similar to lionfish based on diet from Green et al. (2012) and FishBase.

| **Family** | **Species Name** |
| --- | --- |
| Aulostomidae | *Aulostomus maculatus* |
| Carangidae | *Alectis ciliaris* |
| Carangidae | *Carangoides ruber* |
| Carangidae | *Caranx bartholomaei* |
| Carangidae | *Caranx crysos* |
| Carangidae | *Elagatis bipinnulata* |
| Lutjanidae | *Lutjanus analis* |
| Lutjanidae | *Lutjanus apodus* |
| Lutjanidae | *Lutjanus cyanopterus* |
| Lutjanidae | *Lutjanus jocu* |
| Lutjanidae | *Lutjanus mahogoni* |
| Lutjanidae | *Lutjanus synagris* |
| Scorpaenidae | *Scorpaena plumieri* |
| Serranidae | *Cephalopholis cruentata* |
| Serranidae | *Cephalopholis fulva* |
| Serranidae | *Epinephelus adscensionis* |
| Serranidae | *Epinephelus guttatus* |
| Serranidae | *Epinephelus itajara* |
| Serranidae | *Epinephelus morio* |
| Serranidae | *Epinephelus striatus* |
| Serranidae | *Mycteroperca bonaci* |
| Serranidae | *Mycteroperca interstitialis* |
| Serranidae | *Mycteroperca tigris* |
| Serranidae | *Mycteroperca venenosa* |

*Supplemental Figures and Captions*

**
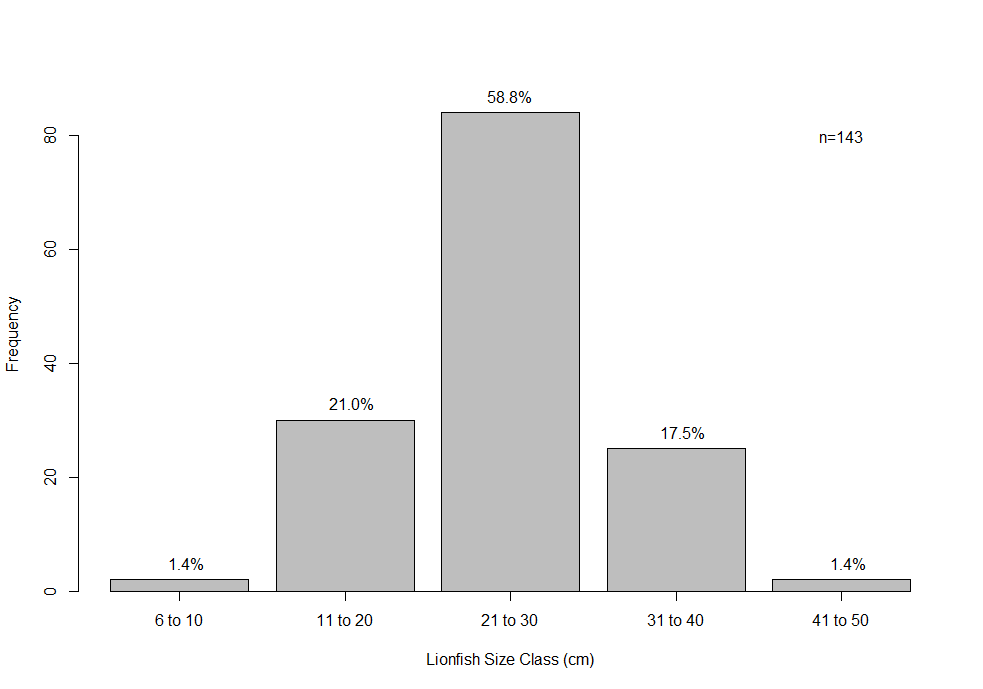
**

**Figure S1. Size Frequency of Lionfish Size Classes.** The frequency of lionfish in each size class (cm) and the percent of the total number of lionfish (n=143).


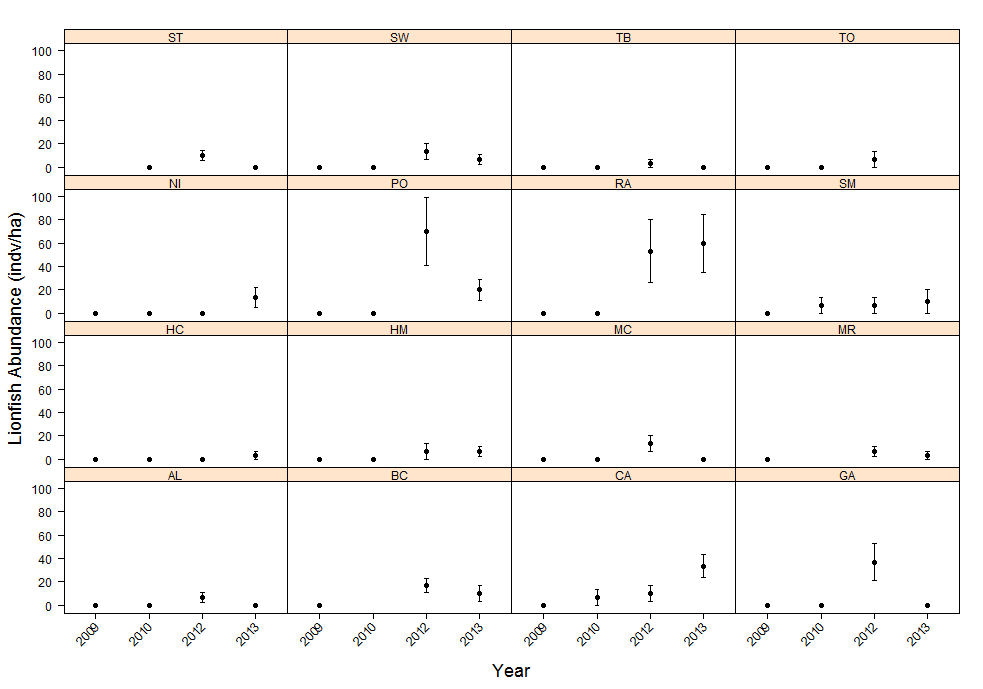


**Figure S2.** **Average Lionfish Abundance from 2009 to 2013 on Each Site.** Lionfish abundance (individuals/ha) over survey years on each survey site.


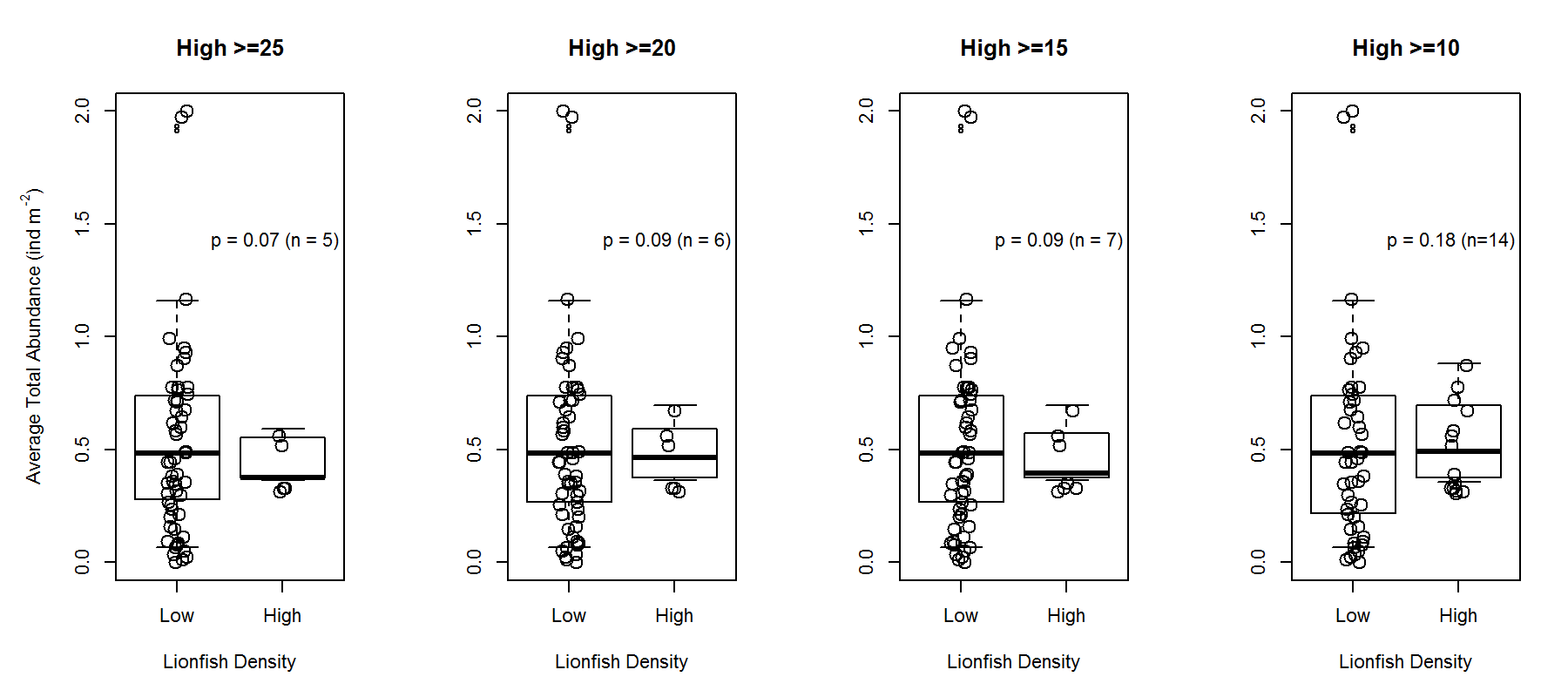


**Figure S3 Abundance of Small Prey Fish in “High” and “Low” Lionfish Densities.** The abundance of small prey fish (6-10 cm TL) split by different threshold values based on published native densities (25-10 lionfish/ha) to determine high vs low lionfish densities. Points are site-level data with medians represented by thick horizontal bars. P-values are from GLMM models with lionfish density was included as a categorical variable. The number of data points in the “high” lionfish density category (n) are displayed for each threshold value.


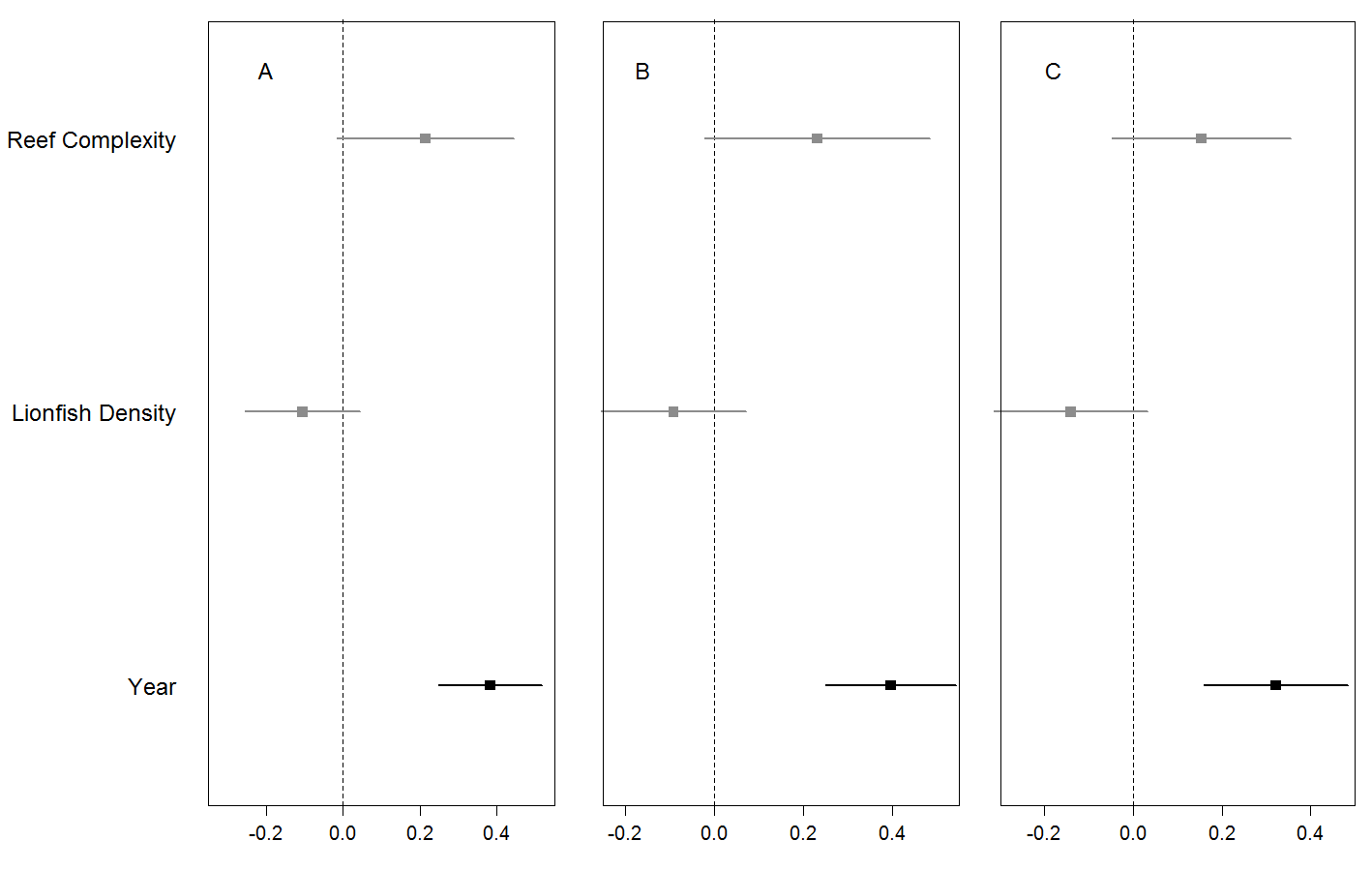


**Figure S4** **Coefficient Estimates** (mean ± 95% confidence interval) **for Each Model of Prey fish Abundance with Different Size Classes.** The site-averaged abundance of potential prey fish of different size classes, 0-10cm TL (A), 0-5cm TL (B), and 6-10cm TL (C), were each modeled with the predictors of interest (lionfish abundance and years since the lionfish invasion) as well as site-specific reef complexity. Significant coefficient estimates are shown in black while non-significant coefficients are shown in gray (details in Table S4).


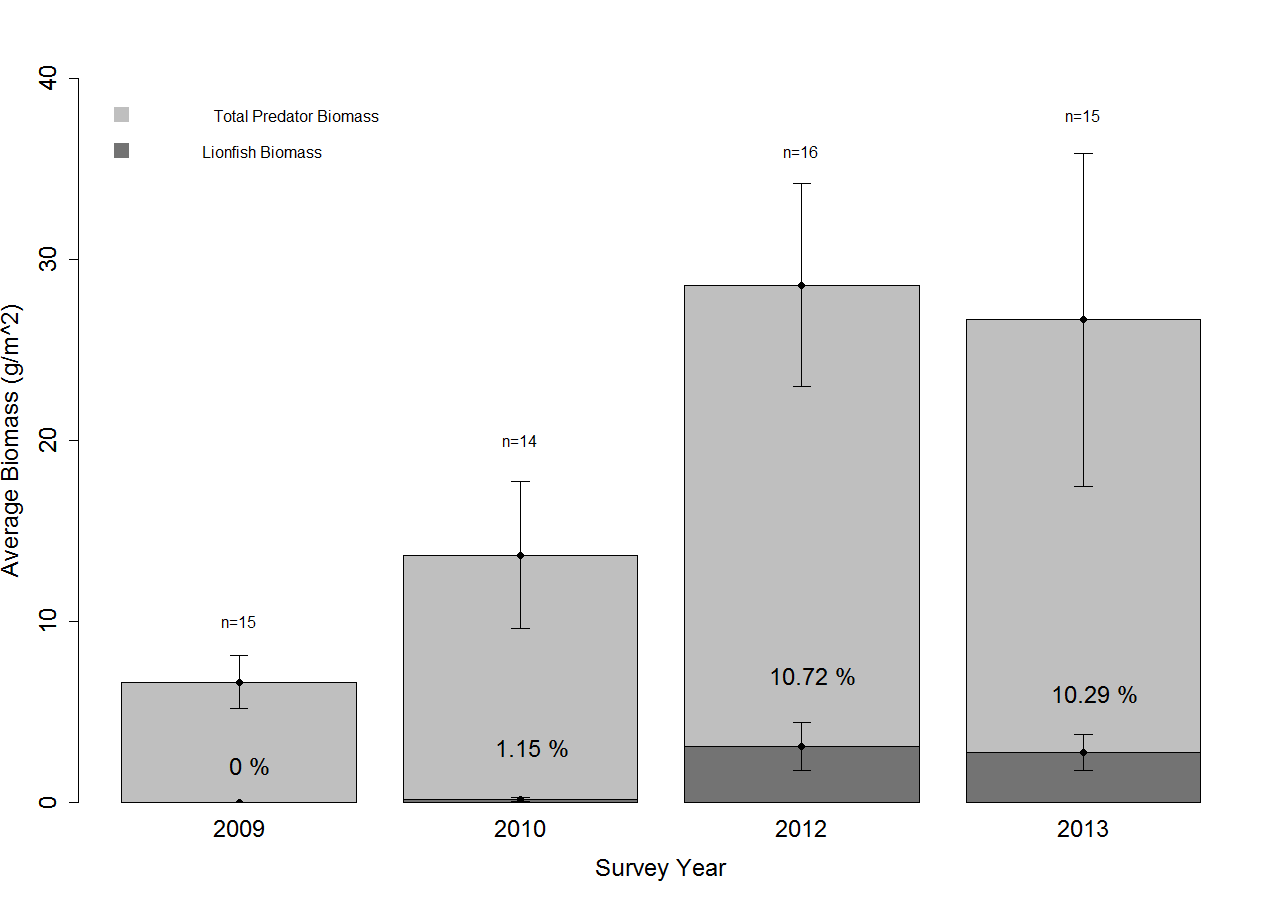


**Figure S5. Biomass of Lionfish and Native Competitors from 2009 to 2013.** The biomass (g/m^2^) of lionfish is shown in light gray, and the total biomass of all ecologically similar native piscivores (see Text S1 and Table S4) is shown in dark gray, from 2009-2013, averaged across sites. The percentage of total predator biomass contributed by lionfish is shown above the average biomass of lionfish in each year. N is the number of sites surveyed in each year.
